# Consequences and predisposing factors of self-discharge against medical advice in plastic and hand surgery

**DOI:** 10.1007/s00423-021-02248-z

**Published:** 2021-08-25

**Authors:** Sören Könneker, Rosalia Luketina, Stefaniya Bozadzhieva, Thomas von Lengerke, Nicco Krezdorn, Theodore L. H. Luketina, Peter M. Vogt, Alexander Kaltenborn

**Affiliations:** 1 Department of Plastic, Aesthetic, Hand, and Reconstructive Surgery, Hannover Medical School, Carl-Neuberg-Str. 1, 30625 Hannover, Germany; 2 Department of Medical Psychology, Hannover Medical School, Hannover, Germany; 3 Department of Anesthesiology, University Hospital Zürich, Zürich, Switzerland

**Keywords:** Self-discharge, Patient autonomy, Informed decision model, Shared decision-making, Compliance, Adherence

## Abstract

**Purpose:**

Therapeutic success of surgical interventions is significantly affected by patients’ adherence. Patient autonomy can lead to unreasonable behavior. We analyzed the consequences and predisposing factors of patient self-discharge in a plastic and hand surgery cohort.

**Study design and setting:**

Data was collected retrospectively in a case–control study with *n* = 73 patients who had self-discharged in a 10-year time period and *n* = 130 controls (discharge by the surgeon). Data was collected through the hospital information systems and a particular questionnaire. Statistical analyses were performed via chi-squared test and logistic regression analyses.

**Results:**

Patients who self-discharged against medical advice had a significantly higher complication rate (p = 0.045) and a higher number of revision operations (*p* < 0.001). They were more often dissatisfied with the primary inpatient treatment (*p* < 0.05). Secondly, they lived more often in shared households (*p* = 0.002; OR 5.387 (1.734–16.732)) or had to take care of their children at home (*p* = 0.006; OR 1.481 (1.280–1.741)). There was a significantly lower pain score (NAS) on time of self-discharge (*p* = 0.002) as well as 24 h after self-discharge (*p* < 0.001) in self-discharged patients.

**Conclusion:**

Self-discharge was associated with predisposing factors and poorer outcomes. Patient autonomy can lead to health-compromising behavior and patients should be counseled accordingly.

## Introduction

The outcome of surgery as well as other healthcare interventions strongly depends on patients’ compliance [1–3]. The World Health Organization (WHO) defined compliance as the extent to which a patient’s behavior corresponds with recommendations from a healthcare provider [4].

Involving the patient comprehensively into the process of decision-making is well described for oncological therapy, where patient satisfaction and quality of life during and after treatment represent essential goals of medical treatment [5]. In general, physician–patient interaction and communication have changed from a paternalistic model, where the physician acts as the dominant decision-maker, to a shared decision-making model (SDM) with patients as active partners, while patient autonomy is maximized in the informed decision model [6]. Whereas it has been shown that SDM improves patient adherence, patient autonomy can lead to unreasonable self-harming behavior, i.e., non-compliance [7, 8]. Though respecting patient autonomy, some authors automatically infer non-compliance from self-discharge [9], while others criticize this negative framing of self-discharge, and believe that discharge against medical advice can be viewed positively [10].

Over the last 10 years, we have observed an increasing number of patient decisions contrary to medical recommendations at our clinic, including self-discharge against medical advice. In order to get a better understanding of patients’ decision-making and improve handling of self-discharge, we set out to analyze consequences and predisposing factors associated with patient self-discharge against medical advice in our department of plastic, aesthetic, hand and reconstructive surgery.

## Study design and setting

This study was carried out as a single-center, non-matched case–control study. The two hospital information systems (SAP, Walldorf, Germany, and DOIT, Meierhofer AG, Munich, Germany) used at our clinic were screened for self-discharged patients in a 10-year period between 2007 and 2017. Three hundred thirty-nine patients were identified for this time period, out of which 73 patients (21.5%) could be followed-up and consented to participate in the study. They were asked to fill out a questionnaire including self-report of reoperations and complications. Data was divided into four subgroups: hand, infection, burn, and others. As a control group, 130 out of 148 regularly discharged patients were included directly at time of discharge in a time period of 40 days in 2017. To include early complications, patient response was expected no earlier than 2 months after treatment. Inclusion criteria for the control group were age over 18 years, inpatient treatment comparable to the study group and discharge by the surgeon.

In addition to the analysis of descriptive parameters, inferential statistics were performed to identify relevant differences between the cohorts. Categorical variables were analyzed with the Pearson’s chi-squared test. A p-value < 0.05 was defined as significant. For the identification of risk factors for self-discharge and complications, univariate multiple logistic regression analyses were performed. All variables with a p-value < 0.05 in univariate analyses were included into the respective multiple logistic regression.

Ethics approval was obtained from the local ethics committee under approval number 2556–2015. All participants filled out the questionnaire including written or verbal informed consent in data processing.

## Results

**Fig. 1:**
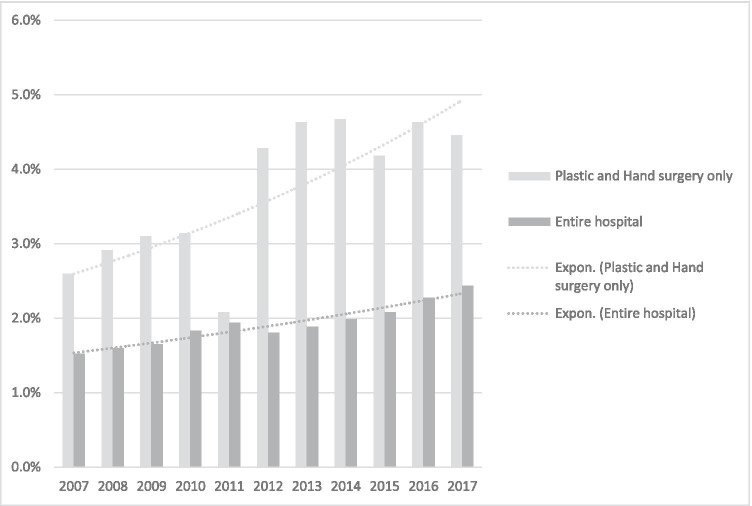
Part of patients with self-discharge against medical advice

The percentage of patients who self-discharged increased from 2.6 to 4.7% over 10 years. This proportion was larger when compared to that of inpatient cases with self-discharge of all other medical disciplines of our hospital (Fig. 1).

An overview of the questionnaire data is shown in Table 1. Patients in the self-discharged group lived in 84.9% of cases in shared households. This background was significantly less prevalent in the control group (67.7%; p = 0.002). This difference was controlled with multivariate analysis (OR 5.387 (95% CI: 1.734–16.732); see Table 2). Furthermore, patients who discharged themselves against medical advice had significantly more children to take care of at home, when analyzed with multivariate analysis (28.8 vs. 23.1%; p = 0.006; OR 1.481 (95% CI: 1.280–1.741); see Table 2).

**Table 1:** Results of the statistical comparison of patients who discharged themselves against medical advice vs. routinely discharged patients

Variable	Discharge type			*p*-value (Pearson’s chi^2^-test)
	Self-discharge against medical advice (*n* = 73)	Discharge by the surgeon (*n* = 130)		
Patient characteristics, predisposing factors, and risk factors				
Hand	38 (52.1%)	50 (38.5%)		0.061
Burn	7 (9.6%)	15 (11.6%)		0.668
Infection	3 (4.1%)	14 (10.8%)		0.100
Others (elective)	24 (32.9%)	51 (39.2%)		0.368
Female	34 (46.6%)	45 (34.6%)		0.094
Unmarried	16 (21.9%)	38 (29.2%)		0.258
Single household	9 (12.3%)	18 (13.9%)		0.760
Children in household	21 (28.8%)	30 (23.1%)		0.370
Shared household	62 (84.9%)	88 (67.7%)		**0.007**
Not working	38 (52.1%)	65 (50.0%)		0.779
Professional qualification	56 (76.7%)	104 (80.0%)		0.582
Treatment was invasive	71 (97.3%)	123 (95.4%)		0.503
Satisfied with treatment	64 (87.7%)	124 (95.4%)		**0.044**
Treatment continued by general practitioner	33 (45.2%)	74 (56.9%)		0.109
Treatment continued by another surgeon	21 (28.8%)	37 (28.5%)		0.963
Treatment continued by outpatient clinic of same hospital	20 (27.4%)	13 (10.0%)		**0.001**
Pain score (NAS) at time of discharge	Mean: 1.8 (SD: 2.4)	Mean: 2.7 (SD: 2.4)		**0.002**
Consequences				
Pain score (NAS) 24 h after discharge	Mean: 1.7 (SD: 2.3)		Mean: 2.7 (SD: 2.4)	** < 0.001**
Complications	14 (19.2%)	13 (10.0%)		0.065
Revision operation	13 (17.8%)	4 (3.1%)		** < 0.001**

Entries in bold indicate statistical significance

**Table 2:** Results of multiple binary logistic regression analysis for the identification of predisposing factors for the outcome “self-discharge against medical advice”

Variable	*p*-value	Odds ratio (95% confidence interval)
At least one child in the household	**0.006**	1.481 (1.280–1.741)
Shared household	**0.002**	5.387 (1.734–16.732)
Marital status (married vs. unmarried)	0.218	n.a
Professional qualification	0.488	n.a
Employed	0.680	n.a
Female	0.242	n.a

Entries in bold indicate statistical significance

*n.a.,* not applicable

After self-discharge, treatment was continued by general practitioners in 45.2%, by other surgeons in 28.8%, or by our outpatient clinic in 27.4% of cases. In the control group, treatments were continued by general practitioners in 56.9%, by other surgeons in 28.5%, or by our outpatient clinic in 10.0% of cases. There was significantly more continuation of therapy by our outpatient clinic in the self-discharge group (p = 0.001).

While self-discharged patients were mostly satisfied with the medical treatment (87.7%), patients who were discharged by their surgeon were even more often satisfied (95.4%; p = 0.044).

No statistically significant differences were found regarding employment status, professional qualification, or type of treatment (see Table 1).

There was a significantly lower pain score (NAS) on time of self-discharge (1.8/10) when compared to control group (2.7/10; p = 0.002). Twenty-four hours after self-discharge, the pain score remained significantly lower (1.7/10 vs. 2.7/10; *p* < 0.001).

Patients who self-discharged against medical advice had a self-reported complication rate during follow-up of 19.2%, compared to 10.0% when discharged by the surgeon (control group). This finding was statistically significant in the logistic regression analysis which adjusted for socio-demographic variables (p = 0.045; OR 2.604, 95% CI: 1.007–6.731; see Table 3). In case of self-discharge, the revision operation rate was also higher (17.8%) when compared to the control group (3.1%; *p* < 0.001). One out of 73 patients regretted the decision of self-discharge.

**Table 3:** Results of multiple binary logistic regression analysis for the identification of risk factors for the onset of postoperative complications

Variable	*p*-value	Odds ratio (95% confidence interval)
At least one child in the household	0.523	n.a
Shared household	0.074	n.a
Marital status (married vs. unmarried)	0.150	n.a
Professional qualification	0.899	n.a
Employed	0.453	n.a
Female	0.244	n.a
Discharge against medical advice	**0.045**	2.604 (1.007–6.731)

Entries in bold indicate statistical significance

*n.a.*, not applicable

## Discussion

In our study, we wanted to take a closer look at patients with self-discharge against medical advice in our clinic for plastic, aesthetic, hand and reconstructive surgery. We found a slightly increasing rate of self-discharge over the examined time period. The outlier in 2011 might be caused by counting errors due to bridging the two hospital information systems. In comparison, the self-discharge rate of all hospital disciplines was increasing too, however with lower rates throughout.

We compared patients who had self-discharged after treatment in our clinic with a control group. The risk for self-reported complications was about two times higher in case of self-discharge, together with a higher rate of reoperations. Other authors showed that readmission rates of patients after self-discharge can be increased by up to the factor 7 [11]. Taken together with living in a shared household or in one with at least one child as significant predictors of self-discharge, as found in our study, this may indicate that some patients may be unable to maintain a complicated regimen without a strong system of social support and prompts to remind them of what needs to be done, or due to childcare responsibilities. At the same time, about 80% do not indicate any complication in our self-discharge group. This finding implies that self-discharge does not inevitably have to be associated with a worse outcome.

Self-discharge against medical advice of dissatisfied patients can be caused by failures in shared decision-making in surgical disciplines as described by many authors [12]. Studies have found that both patient satisfaction and patient adherence are enhanced by patients’ involvement and participation in their care [13, 14].

Possible causes of patients’ dissatisfaction during hospital stay are not solely associated with medical treatment [15]. Satisfaction with nursing care and satisfaction with organizational features may have an impact on general satisfaction, and also on patients’ decision to self-discharge. These causes tend to be insufficiently considered in the existing literature about self-discharge. This is demonstrated by the higher rate of patients we observed who came back to continue the treatment as an outpatient in our clinic. Eventually, however, in our study, we were not able to grasp all aspects of causes for dissatisfaction.

There was a lower indicated pain score in patients at time of self-discharge compared to control group. However, this factor did not lead to patients’ staying in house. One explanation may be that pain is only one component of dissatisfaction, and other factors which lead to self-discharge may have a stronger impact on patient’s decision.

Limitations of this study should be considered. Our study design was retrospective, which is prone to bias in general. Patients who completed the questionnaire had to remember their previous inpatient and later outpatient treatment. Although this is a general problem in outcome measurements via questionnaires, it could especially make distortions in our study because of different periods between treatment and survey. Nevertheless, we found no differences when surveys of long and short periods were compared. Due to the study design and the different continuations of hospital treatment as described above, details of the postoperative course are necessarily limited. Furthermore, we employed an unmatched pair case–control design, which was due to difficulties in recruiting enough self-discharging patients at the time of self-discharge. The attempt was given up because patients who discharged themselves were unwilling to sign the consent to participate. Maybe this point was due to fear of consequences, embarrassment, or other circumstances owed to the situation at time of self-discharge, as patients were not addressable for additional effort. However, less than 10% of patients asked to participate (only 3 out of 40) signed the consent. Therefore, we decided to request self-discharged patients retrospectively and achieved a positive response in more than 20% of requested patients.

The invasiveness of initial surgery was not considered and compared between the two groups; however, this aspect should have been adjusted for by the surgeon’s advice for further inpatient treatment. At the same time, between the four subgroups: hand, infection, burn, and other statistically significant differences were not found. Finally, due to a data management error, age was not available as variable for approximately half of the included patients, which is why it had to be omitted in the present analysis. However, results of regression analyses for patients for which the variable was available did not differ from those reported above.

In summary, we found higher complication rates as possible consequences of self-discharge against medical advice. We also found some predisposing factors like potential support (shared households) or responsibilities (childcare) at home. In addition to these understandable reasons for premature termination of inpatient treatment, patient’s dissatisfaction and ignorance about consequences might present a lack of communication in shared decision-making for some of the patients. Without detailed explanation of the rational reasons for further inpatient treatment, patients can perceive their inpatient treatment as an unnecessary hospital stay. However, delay in discharge management, for example, due to a lack of medical staff, might be an understandable motivation for impatient patients to discharge themselves. A realistic assessment of patients’ knowledge and understanding of the regimen, and their trust in it, will enable a more effective targeting of potential compliance problems. At the same time, the physician–patient partnership itself remains at the core of all successful attempts to improve adherent behaviors.

## Data Availability

Data available within the article and additional data available upon request.
